# Tumor size and effectiveness of electrochemotherapy

**DOI:** 10.2478/raon-2013-0002

**Published:** 2013-02-01

**Authors:** Barbara Mali, Damijan Miklavcic, Luca G. Campana, Maja Cemazar, Gregor Sersa, Marko Snoj, Tomaz Jarm

**Affiliations:** 1 Faculty of Electrical Engineering, University of Ljubljana, Ljubljana, Slovenia; 2 Sarcoma and Melanoma Unit, Veneto Region Oncology Research Institute (IOV-IRCCS), Padova, Italy; 3 Institute of Oncology Ljubljana, Ljubljana, Slovenia

**Keywords:** electrochemotherapy, cutaneous tumors, effectiveness, tumor size, meta-analysis

## Abstract

**Background:**

Electrochemotherapy (ECT) is an effective and safe method for local treatment of tumors. However, relatively large variability in effectiveness of ECT has been observed, which likely results from different treatment conditions and tumor characteristics. The aim of this study was to investigate the relationship between tumor size and effectiveness of a single-session ECT.

**Materials and methods:**

A systematic search of various bibliographic databases was performed and nine studies eligible for this study were extracted. Different statistical methods including meta-analysis were applied to analyze the data.

**Results:**

The results of analysis based on data from 1466 tumors of any histotype show significantly lower effectiveness of ECT on tumors with maximal diameter equal to or larger than 3 cm (complete response (CR) of 33.3%, objective response (OR) of 68.2%) in comparison to smaller tumors (CR% of 59.5%, OR% of 85.7%). The results of meta-analysis indicated that ECT performed on tumors smaller than 3 cm statistically significantly increases the probability of CR by 31.0% and OR by 24.9% on average in comparison to larger tumors. The analysis of raw data about the size and response of tumors showed statistically significant decrease in effectiveness of ECT progressively with increasing tumor diameter. The biggest drop in CR% was detected at tumor diameters as small as 2 cm.

**Conclusions:**

The standard operating procedures for ECT should be reexamined and refined for the treatment of large tumors. We propose that future clinical trials should include accurate ECT treatment planning and/or multiple ECT cycles, besides a prolonged observation for tumor response evaluation.

## Introduction

Treatment of cutaneous and subcutaneous tumors using electrochemotherapy (ECT) has gained its role in routine clinical practice. The reason for an increasing use of ECT in clinics arises from favorable treatment characteristics, which are high effectiveness, safety, simplicity, low toxicity, possible application in an outpatient setup and cost-effectiveness. 1–7 The standard operating procedures (SOP) for ECT using the Cliniporator device were prepared during the European Standard Operating Procedures of Electrochemotherapy (ESOPE) project. 1,8 The aim of the SOP document was to define guidelines for safe and effective ECT of cutaneous and subcutaneous tumors. Different treatment procedures were proposed within the SOP with respect to the number, size (maximal diameter) and depth of tumors. The SOP document was developed based on the experience from the leading European cancer centers using ECT, and tested during the ESOPE project in which also tumors larger than 3 cm in diameter were treated but they were excluded from ECT treatment evaluation reported in ESOPE study.^1^ More recently, some researchers apply ECT also for the treatment of tumors larger than 3 cm.^9^–^11^ Although general recommendations for ECT procedures on large tumors are given in the SOP, it is unclear whether this recommendations are appropriate for tumors with diameters larger than 3 cm.

The purpose of this study was therefore to examine the relationship between tumor size and tumor response to treatment based on local tumor control of single-session ECT (using merged evidence from different studies) and to address the issue of the SOP for large tumors.

## Materials and methods

### Study selection and data extraction

All steps for a systematic review from PRISMA guidelines were applied in this study.^12^–^14^

The publicly available literature was systematically searched to obtain relevant published articles about clinical evaluation of effectiveness of ECT on tumors of various sizes. The following 16 databases were searched: Web of Science, Science Direct, PubMed, Wiley Online Library, OvidSP, HighWire Press, IEEE Xplore, SpringerLink, nature. com, Compendex, BioMed Central, Ingenta, Inspec, Journal Storage, The Cochrane Library, and Medscape. The search terms “electrochemotherapy” and “clinical” were used and the time span between 1^st^ January 1991 and 22^nd^ November 2011 was considered. Author BM first examined the titles and abstracts of the studies identified with the search strategy to narrow the initial selection of studies and then made the final selection based on full text reading. Authors TJ and GS independently checked the preliminary selection of studies. Bibliographies of original articles, review articles and relevant books were also screened to identify other potentially eligible studies. Articles published electronically were included but abstracts, posters, reviews, editorials, lectures and commentaries were not included in systematic review. In addition, the data collected at the Institute of Oncology Ljubljana (denoted as IO data from this point onwards) was also recognized as appropriate and was hence included in the analysis.

A study was considered eligible for meta-analysis if the following criteria were met:

1. inclusion of data for single-session ECT of cutaneous or subcutaneous tumors of any histotype performed on human patients;
2. inclusion of data about number of patients and tumors, size and response of tumors, histotype of tumor; electrode type, drug type and route of administration;
3. response of tumors evaluated at least 4 weeks after ECT treatment according to WHO or RECIST criteria, or with diagnostic imaging or biopsy;^15^,^16^
4. data about size and response of tumors was reported in such a way that separation of tumors into two groups was possible: tumors with maximal diameter smaller than 3 cm and tumors with maximal diameter equal to or larger than 3 cm.

The cutoff dimension of tumor size of 3 cm in the last (fourth) criterion was selected because the majority of studies included in data analysis reported data of tumor responses only for group of tumors smaller and equal to or larger than 3 cm without details that would allow using a different cutoff value. The custom cut off value can be set only for two studies with full access to raw data (IO data and data from Campana *et al.*^9^).

The following data was extracted from eligible studies by two of the authors (BM and TJ) independently: author and year of publication, number of patients, number, size and response of tumors, tumor histotype, electrode type, chemotherapeutic drug and route of its administration, criteria for tumor response evaluation, duration of follow-up and assessment of risk of bias of the study. Differences in extracted data between both authors were discussed to find the source of disagreement and to reach a common final decision. If the same data was used in two or more studies, either the first published or the more comprehensive study was included in the analysis. Authors of three studies included in the analysis were contacted for additional data, which were not included in published articles, but were needed for this study.^9^–^11^

The risk of bias of the studies was assessed following the Cochrane Collaboration recommendations. 14 Ratings for each bias issue (low, high or unclear bias) were extracted independently by two authors (BM and TJ) who were not blinded to names of the authors or locations of the studies. Ratings of both authors were compared and dissimilarities were discussed until consensus was reached. Studies were further rated as having an overall low (all bias issues rated with low), high (any bias issue rated with high) or unclear risk of bias (no bias issue rated with high and any bias issue rated with unclear).

In this study, the tumor response to a single-ECT application was evaluated. The tumor response was classified as either complete response (CR), partial response (PR), no change (NC) or progressive disease (PD), according to the response criteria adopted in the studies (WHO or RECIST), or the pathologic response, assessed by biopsy.^15^,^16^ Although WHO and RECIST criteria are different in some respects, these criteria are essentially equivalent for the evaluation of tumor response on individual lesions (the per-tumor effectiveness), which is the level of response considered in this study. CR is defined as a disappearance of tumor, PR as a decrease of at least 50% in the products of the two largest perpendicular diameters of the tumor (corresponding to tumor area), PD as an increase of more than 25% of lesion area. In all other cases, a response is determined as NC. Tumor response was determined not earlier than 4 weeks post treatment by two observations not less than four weeks apart. Tumors with CR and PR responses were further combined in the so called objective response group (OR) and tumors with NC and PD responses were grouped in the no response group (NR).

### Statistical analyses

The overall effectiveness of ECT was determined across all eligible studies by pooling the response data of individual tumors of all studies together. For this purpose, complete and objective response rate (denoted as CR% and OR% respectively) were calculated across all eligible studies. The same calculations were also performed separately for the group of tumors with maximal diameter smaller and larger than (or equal to) 3 cm. CR% and OR% results of these two groups were compared using two-sided Chi-square test and the difference was considered statistically significant for *p* <0.05.

The CR% and OR% values result in a summary in which all individual tumors from all studies contribute equally. Consequently, the relative contribution of each study to these values is proportional to its relative size. When applying statistical analysis on data accumulated from a series of studies that had been performed by researchers operating independently, it would be unlikely that all the studies were functionally equivalent. In such cases, a meta-analysis based on the random-effects model is generally the preferred method for pooling the results of independent studies.^14^,^17^ By applying meta-analysis we obtained the most reliable estimate of the difference in effectiveness of ECT correlated to tumor size. The software for metaanalysis calculations was written in Matlab following the procedures published in the literature.^14^,^17^ The so-called risk difference (RD) was used as the measure of the effect because of dichotomous nature of tumors’ response data. RD is defined as the probability of response (either CR or OR) in one group minus the probability of the same response in the other group. The between-study heterogeneity was assessed with the I^2^ statistic. The summary effect of meta-analysis was combined using a so-called random-effects model. This model considers the within-study variance and the between-studies variance and as a consequence the confidence interval (CI) of the summary effect is wider than in case of the fixed-effects model (thus requiring a larger difference between the two groups in order to find this difference significant). But by using the random-effects model, the larger studies (with many tumors) are also less likely to dominate the overall effect and smaller studies (with few tumors) are less likely to be trivialized than with the fixed-effects model.^14^,^17^ The difference between the two groups of tumors was considered statistically significant for *p* <0.05.

A sensitivity analysis was applied to investigate the influence of studies of high risk of bias on the overall results of data analysis.

The raw data about the size (maximal diameter of tumor) and response for tumors from article by Campana *et al.* were used for examination of relationship between tumor size and response.^9^ Spearman’s rank correlation coefficient and its significance were used for determination of statistical dependence between these two parameters. The tumors were also grouped by their size into four groups with 1 cm step size with the last group including all tumors equal to and larger than 3 cm, *i.e.* <1 cm, 1–2 cm, 2–3 cm and >3 cm. The differences in proportion of CR, PR and NR were tested between neighbor groups using Chi-square test in order to find the range of tumor size with statistically significant decrease of CR and OR and increase of NR with respect to its neighbor group. The same statistical tests were also performed on IO data. Due to a full access to IO data, the statistical comparisons of additional parameters (tumor area, volume, histotype and location; drug type and route of administration; current, voltage and energy per area delivered on tumor; electrode type; median follow-up) were performed between the groups. Rank Sum test on ordinal data and Chi-square test on nominal data were applied using statistical toolbox in Matlab and the difference was considered statistically significant for *p* <0.05.

## Results

### Study selection and data extraction

**FIGURE 1.**
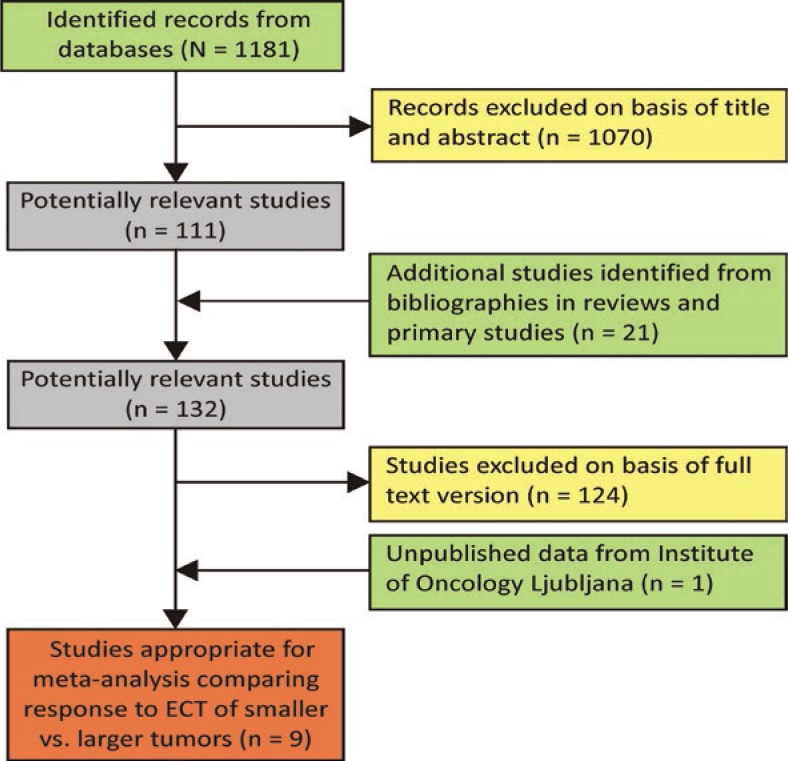
Selection process for the studies included in the data analysis.

The flow chart of the selection process for the studies included in data analysis is given in Figure 1. The initial search of 16 databases resulted in 1181 records after removal of duplicates but finally only eight articles satisfied all criteria.^9^–^11^,^18^–^22^ The IO data from a clinical database of ECT performed on cutaneous and subcutaneous tumors at Institute of Oncology Ljubljana also met all the selection criteria and was therefore included as the ninth study. All these studies were non-randomized phase I or II studies.

The risk of bias (rated as low, high or unclear) was assessed for individual studies included in the analysis (Table 1). No assessment of overall risk of bias was possible for IO data because most of this data has not been previously published. Note that some of these data (about 40% of patients and 25% of tumors) has been published previously in the ESOPE study.^1^

The characteristics of the studies used for systematic review are shown in Table 2. In total, 1466 tumors and 197 patients were included. There were 252 (17.2%) tumors with maximal diameter larger than or equal to 3 cm, and 1214 (82.8%) tumors smaller than 3 cm.

### Statistical analyses

Overall CR% and OR% of 55.0% and 82.7% were determined respectively, across all included studies irrespective of tumor size (Table 2). The results show higher effectiveness of ECT on tumors with the largest diameter smaller than 3 cm (CR% and OR% of 59.5% and 85.7%, respectively) in comparison to tumors with the largest diameter equal to 3 cm or larger (CR% and OR% of 33.3% and 68.2%, respectively). The differences of CR% and OR% between these two groups of tumors were statistically significant, both with *p* <0.001. Consequently, the proportion of tumors with NR (combining the cases of NC and PD after a single application of ECT) was statistically significantly higher for the larger tumors’ group in comparison to the smaller tumors’ group (NR% of 31.8% and 17.3%, respectively, *p* <0.001).

**FIGURE 2.**
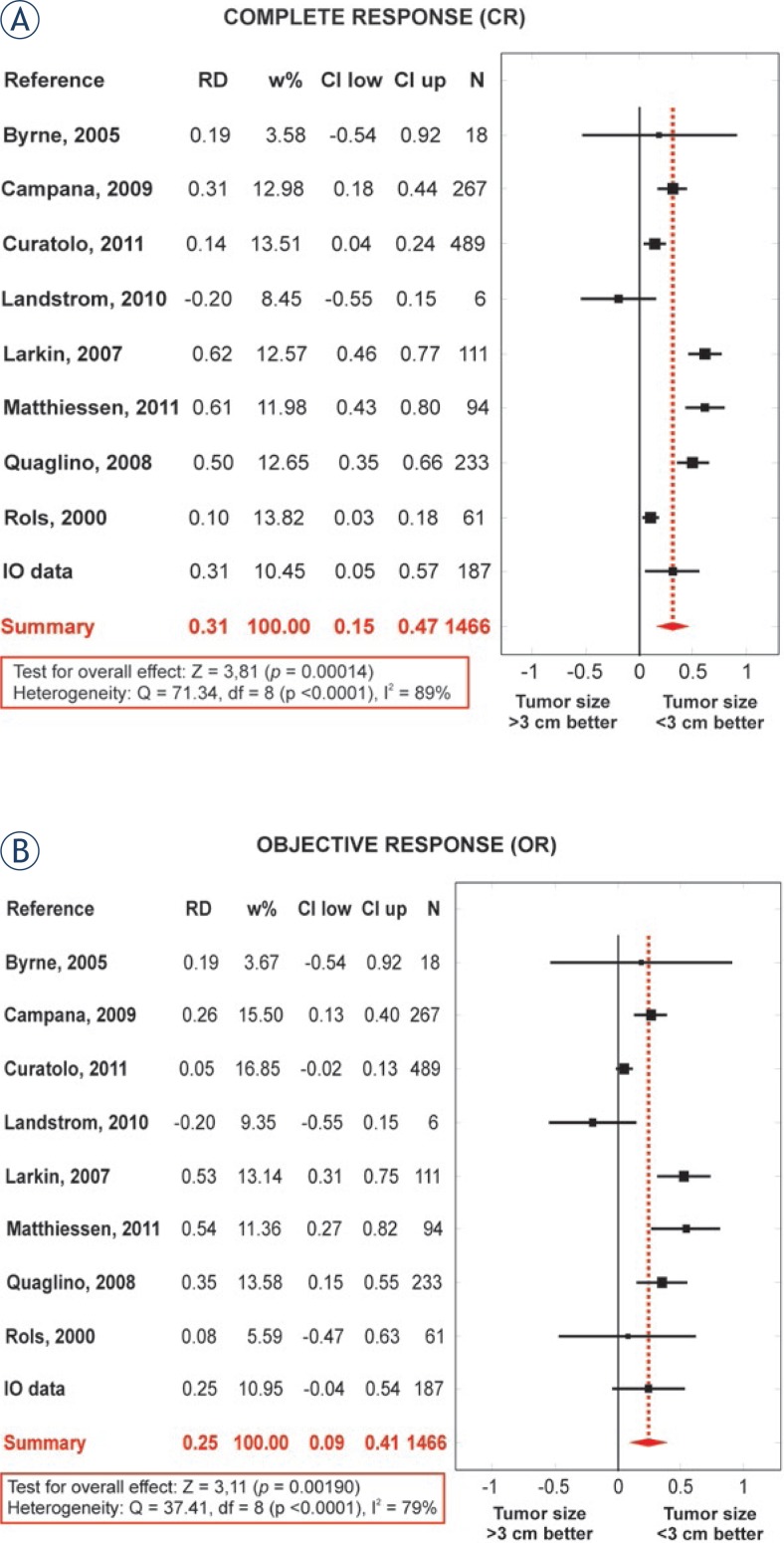
Results of meta-analysis. Data for individual studies and pooled results (Summary) demonstrating: (A) a statistically significant 31% increase in probability of CR for tumors smaller than 3 cm in comparison to tumors equal to or larger than 3 cm with ECT, and (B) a statistically significant 25% increase in probability of OR for tumors smaller than 3 cm in comparison to tumors equal to or larger than 3 cm with ECT. RD = individual and summary risk difference for studies included in meta-analysis; w% = weight of study in comparison to all studies; CI low and CI up = the lower and upper confidence interval of RD, respectively; N = the number of tumors per each study and total number of tumors included in meta-analysis

Similarly, the results of meta-analysis demonstrated that ECT performed on tumors smaller than 3 cm increases the probability of CR and OR by 31.0% and 24.9% on average, respectively, in comparison to tumors equal to or larger than 3 cm (summary RD values, see Figure 2). The results of summary risk difference (RD) for CR and OR were statistically significant with significances of <0.001 and 0.002, respectively.

For the sensitivity analysis, two studies (Rols *et al.*, 2000 and Campana *et al.*, 2009) with an overall rating of high risk of bias were removed from the statistical analysis to see if overall results are affected by the inclusion of studies with high risk of bias.^9^,^22^ These two studies accounted for 22.4% of all tumors included in this study. The differences in CR% and OR% between groups of tumors of different size both remained statistically significant with *p* <0.001 (CR% and OR% of 62.5% and 87.9% respectively for smaller tumors, CR% and OR% of 35.4% and 70.8% respectively for larger tumors). When compared to CR% and OR% values in Table 1, the change of these values was relatively small in comparison to the variability of results between different studies. Similarly small changes in results were also found for meta-analysis when studies with a high risk of bias were excluded. Namely, RD for CR of 0.34 (CI between 0.13 and 0.55) and RD for OR of 0.26 (CI between 0.05 and 0.46) were obtained (compare these values to data in both summary lines of Figure 2). Both results for CR and OR however remained statistically significant with *p* = 0.002 and *p* = 0.015, respectively.

**FIGURE 3.**
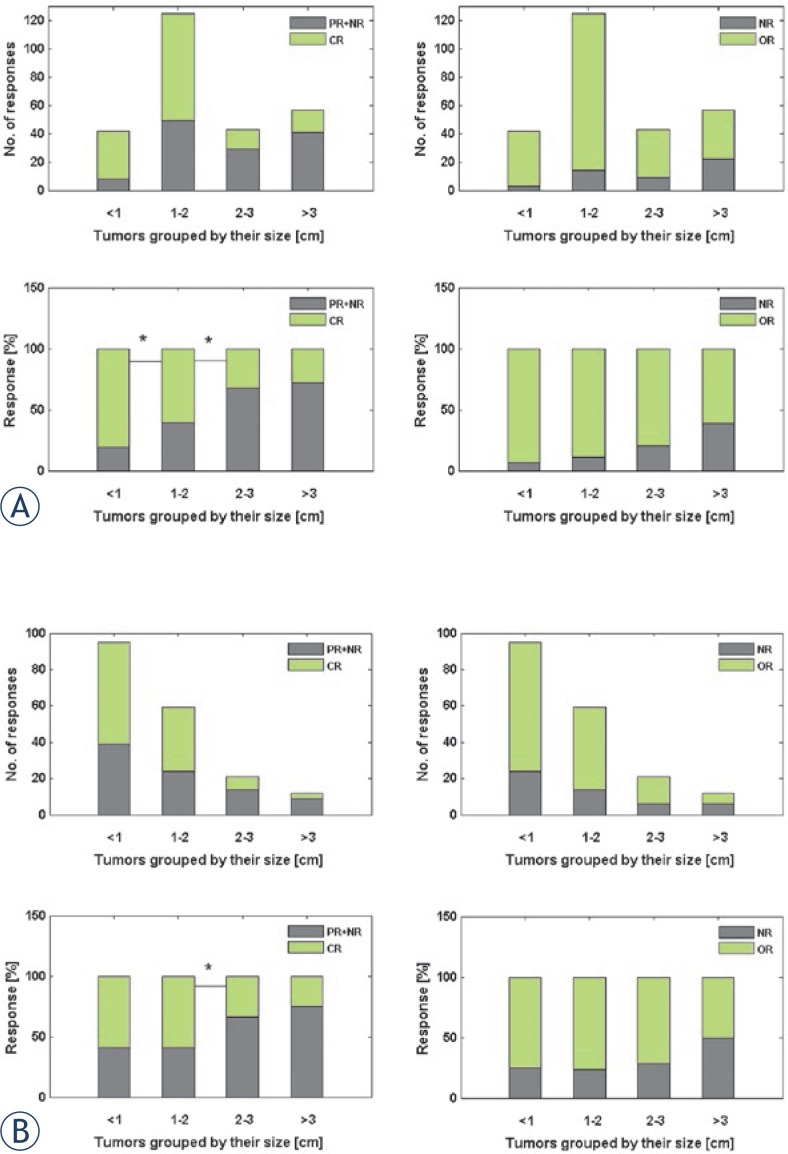
Number and proportion of tumor CR and OR to ECT with respect to tumor size for data: (A) from Campana *et al* and (B) from unpublished IO data. Tumors were grouped by their size using a 1 cm step. Each pair of neighbor groups, for which a statistically significant difference in proportion of CR and OR was found, is indicated with *. OR = objective response; CR = complete response; PR = partial response; NR = no response

The analysis of raw data for the size and response for tumors from the study by Campana *et al.* showed that the effectiveness of ECT, defined as CR%, was decreasing progressively with increasing maximal tumor diameter (Spearman’s rho = 0.418, *p* <0.001) (Figure 3A). The statistically significant drop in CR% (but not significant drop in OR% and increase in NR%) was detected between group of tumors of size <1 cm and 1–2 cm (*p***=** 0.017), as well as between group of tumors of size 1–2 cm and 2–3 cm (*p***=** 0.001), where the most evident drop in CR% was detected.

Similar results were obtained for the IO data, in which also similar tendency of decrease in effectiveness of ECT (expressed as CR%) with increasing size of treated tumors was detected (Spearman’s rho = 0.129, *p*= 0.078) (Figure 3B). The maximal drop in CR% was detected between group of tumors of size 1–2 cm and 2–3 cm, and was statistically significant with p = 0.041. Due to full access to IO data, we were able to investigate if there was some other parameter beside the tumor size (such as tumor histotype and location; drug type, dose and route of administration; current, voltage and energy per area delivered on tumor; electrode type; median follow-up) that could be correlated with the observed difference in tumor response between these two size groups of tumors. Based on statistical comparison, these two groups of tumors of size 1–2 cm and 2–3 cm proved to be imbalanced with respect to upper listed parameters; therefore, no other parameter that would correlate with the difference in tumor response between these two size groups of tumors could be found. Among them, significant imbalance in proportion of melanoma and non-melanoma tumors, and drug type and route of administration used was identified.

## Discussion

The main prerequisites for an effective ECT treatment are an adequate extracellular concentration of the chemotherapeutic drug in the entire tumor at the time of pulse delivery and the coverage of tumor volume with an electric field able to permeabilize the cell membrane and therefore to enable drug uptake. 23–26 Sufficiently high electric field in the tumor tissue can be assured by delivery of pulses of adequately high voltage and appropriate positioning of the electrodes. In addition, some other conditions or parameters could be relevant, such as patient and tumor (histotype, size and location) characteristics and treatment parameters (drug, dose and route of administration, electrode type, protocol and timing of pulse delivery). In this study, we investigated the correlation between tumor size and effectiveness of ECT. Individual tumor data were gathered from heterogeneous non-randomized studies with various levels of additional information available; therefore we were not able to assess the possible cause-effect relationship between other parameters and the treatment response.

Our results showed that ECT was less effective on tumors larger than 3 cm in comparison to tumors smaller than 3 cm (CR% and OR% of 59.5% and 85.7%, respectively, versus CR% and OR% of 33.3% and 68.2%, respectively, Table 2). On the other hand, the no response rate (NR%) had more than doubled on larger tumors when compared to NR% on smaller tumors (from 14.3% to 31.8%, Table 2). The results of meta-analysis confirmed these findings, by showing that the effectiveness of ECT on the smaller tumors was significantly higher than on the larger ones, when the size limit between smaller and larger tumors was set to 3 cm (Figure 2) regardless of large heterogeneity of the included studies (Table 2). All results remained statistically significant when studies with an overall high risk of bias were excluded. The sensitivity analysis thus showed that the overall results and conclusions are not affected by the inclusion of studies with high risk of bias. Therefore, our results can be considered with a higher degree of certainty.

The trend of decreasing ECT effectiveness with the increasing tumor size was clearly demonstrated with the analysis of raw data derived from the paper by Campana *et al.* and confirmed by the analysis of unpublished data from Institute of Oncology Ljubljana (IO data) (see Figure 3).^9^ The results of the analysis based on the data from these two independent sources (the only two available for more detailed analysis) revealed that proportion of CR% was statistically significantly decreased already for tumors with maximal diameter around 2 cm (see Figure 3).

When treating large tumors with ECT, the SOP document suggests the administration of bleomycin by the intravenous route and use of needle electrodes in order to cover the whole tumor with sufficiently high electric field.^8^ Almost all studies included in our survey were conducted according to the SOP recommendations, except for the study by Byrne *et al.* in which only intratumorally administered bleomycin was used and the study by Rols *et al.* in which strictly plate electrodes were used (Table 2).^18^,^22^ Both these studies predate the publication of the SOP. Even though the SOP recommendations were generally followed in the studies included in the analysis, a relatively low response rates (CR% or OR%) were obtained in ECT treatment of tumors larger than 3 cm.

The first possible explanation for decreased effectiveness of ECT in tumors larger than 3 cm that should be considered is inadequate concentration of chemotherapeutic drug reached in the target tumor due to improper timing of pulse delivery. In the analyzed studies, pulses were applied either around 2 minutes after intratumoral bleomycin or cisplatin administration (which is within 10 min after drug administration as recommended in SOP), or within the therapeutic window of 8–28 minutes after intravenous bleomycin administration. 8,23 The interval between intratumoral drug administration and pulse delivery is adequate according to study by Cemazar *et al*.^27^ The “optimal” therapeutic window for intravenous bleomycin administration (originally proposed by Domenge *et al.*) was actually determined based on data from a single patient on whom the ECT was performed in two sessions.^23^ But according to the study by Front *et al.*, the concentration of intravenously administered bleomycin in interstitial fluid around tumor is high enough for efficient ECT treatment for considerably longer period after the injection than the “optimal” therapeutic window recommended within the SOP.^28^ Plasma concentration of bleomycin declines biexponentially with a mean distribution half-life of approximately 24–30 min and mean elimination half-life of 2–4 hours,^29^–^31^ which means that bleomycin concentration within tumors declines relatively slowly in the first two hours after intravenous administration. Therefore, if the insufficient extracellular drug concentration in tumors was indeed responsible for the demonstrated lower effectiveness of ECT on tumors larger than 3 cm, it is very unlikely that it happened due to missed optimal therapeutic window for application of pulses. Nevertheless, further studies are needed to re-examine the current SOP recommendations for “optimal” treatment window. A more appropriate definition of the “optimal” therapeutic window should probably take into account other factors such as histotype, size and anatomical location of tumors to be treated, in addition to the drug type and time and route of its administration.

The second very likely reason for reduced effectiveness of ECT in large tumors is the insufficient exposure of the tumor to the drug, due to heterogeneous distribution of blood flow. It was reported that the periphery of the tumor is considerably better perfused than the inner portion, thus suggesting that the concentration of the drug in the center of the tumor can be lower than in the periphery of the tumor.^32^–^34^ In addition, large temporal and spatial heterogeneity in blood flow is typical for tumors.^35^ Higher drug concentrations in the inner portion of large tumors could be achieved by an appropriate combination of both intratumoral and systemic (intravenous) administrations.

The third possible explanation for the lower effectiveness of ECT in large tumors might be the insufficient coverage of the entire tumor volume with sufficiently high electric field. To overcome this problem, an individualized treatment planning based on radiological imaging could be adopted to determine the appropriate voltages based on the size, geometry and electrical properties of the target region.^36^–^39^ Another option to maximize the tumor response could be to perform ECT treatment with fixed-geometry electrodes and their multiple and overlapped insertions.

The timing of response evaluation after ECT treatment for tumors should also be taken into consideration when interpreting the results of clinical studies. In this study, we considered tumors whose response assessment was performed at least 4 weeks after ECT, according to the SOP document.^8^ However, longer healing time can be expected for larger tumors and 4 weeks after ECT may be too soon for evaluation of the response to ECT in many if not all large tumors. A healing time for smaller tumors is expected to be between 4 and 8 weeks, whereas for larger tumors (larger than 1.5 cm) can be prolonged to up to 10 weeks.^8^ In this study, it turned out that 7 out of 9 studies reported response of tumors at least 8 weeks after ECT treatment. The remaining two studies (Rols *et al.*^22^ and IO data) reported response evaluated less than 8 weeks after treatment only for small portion of tumors. If tumors with the response evaluated earlier than 8 weeks after ECT are not included into analysis, the results remain practically identical and the conclusions of this study remain unchanged. Nevertheless, for more accurate assessment of correlation between tumor size and response, longer follow-up observations should probably be more appropriate, especially because in general the kinetics of response of various tumors after ECT is unknown.

In this study, we considered exclusively the effect of a single-session of ECT. However, several clinical studies reported that the result of ECT on large tumors can be improved with repetitive treatments. 9,11 Moreover, ECT retreatment is not only recommended to achieve a better response in case of larger tumors, but also for smaller tumors unresponsive to the first ECT treatment.

In addition to investigation of correlation between tumor size and response, we intended to evaluate the influence of other tumor and treatment parameters on effectiveness of ECT (tumor area, volume, histotype and location; drug, dose and route of administration; current, voltage and energy per area delivered on tumor; electrode type; median follow-up). Such multivariate data analysis was unfortunately not possible due to unavailability of the details concerning these parameters for individual tumors in the analyzed studies. With such data reported in future clinical reports or with initiated ad hoc study, the reliable estimation of the most important influential parameters on response of large tumors will become possible.

In conclusion, the response of large tumors to ECT treatment seems not to be as good as that reported in smaller tumors. Tumor size starts to play a significant role in the final treatment outcome for tumors as small as about 2 cm in diameter. Therefore, we suggest that the SOP should be refined to improve the effectiveness of ECT for larger tumors. The optimal way to treat larger tumors should include individualized treatment planning to determine the appropriate electrode geometry and voltages, or, alternatively, the application of fixed-geometry electrodes with their accurate repositioning in order to overlap the treated volumes. Moreover, bleomycin could be administered combining both the intravenous and the intratumoral routes to achieve sufficient extracellular concentration in the portion of the tumor. The possibility of repetitive treatments on large tumors (already introduced in the clinical practice in some centers^9^,^11^) should be explicitly suggested within the SOP document, including the recommended interval between ECT cycles. Finally, for an accurate assessment of the correlation between tumor size and response to ECT in larger tumors, a longer follow-up (at least 3 months) could be required.

## Figures and Tables

**TABLE 1.** Assessment of risk of bias for studies included in the analysis (except for IO data)

**First author, year of publication, reference**	**Adequate sequence generation**	**Allocation concealment**	**Blinding of participants and operators**	**Incomplete outcome data**	**Selective outcome reporting**	**Other bias**	**Overall risk of bias**
**Byrne, 2005**^18^	unclear	unclear	unclear	low	unclear	unclear	unclear
**Campana, 2009**^9^	unclear	unclear	unclear	unclear	high	high	high
**Curatolo, 2011**^10^	unclear	unclear	unclear	low	unclear	low	unclear
**Landstrom, 2010**^19^	unclear	unclear	unclear	low	low	low	unclear
**Larkin, 2007**^20^	unclear	unclear	unclear	low	unclear	unclear	unclear
**Matthiessen, 2011**^21^	unclear	unclear	unclear	low	unclear	low	unclear
**Quaglino, 2008**^11^	unclear	unclear	unclear	unclear	unclear	low	unclear
**Rols, 2000**^22^	high	unclear	unclear	low	high	unclear	high	

**TABLE 2.** Summary of studies eligible for meta-analysis comparing the response to ECT of tumors smaller than 3 cm with tumors larger than 3 cm

**First author, year of publication, reference**	**No. of patients/tumors**		**No. of responses of tumors < 3 cm**				**No. of responses of tumors ≥ 3 cm**				**Drug, route**	**Electrode type, electroporator**	**Histotype of tumor(s)**	**Response evaluation**	**Median follow-up in mo. (range)**

	**All**	**Included**	**OR**	**CR**	**PR**	**NR**	**OR**	**CR**	**PR**	**NR**					
**Byrne, 2005**^18^	19/63	15/18	11	11	0	5	1	1	0	1	bleo, i.t.	needle, Medpulser	melanoma	WHO, biopsy	6 (3–6)
**Campana, 2009**^9^	52/608	52/267	184	124	60	26	35	16	19	22	bleo, i.t. or i.v. or both	needle, Cliniporator	melanoma, breast cancer, sarcoma, SCC, head and neck cancer	RECIST	nd (2–21)
**Curatolo, 2011**^10^	23/532	23/489	330	225	105	38	102	57	45	19	bleo, i.v.	plate or needle, Cliniporator	Kaposi sarcoma	RECIST	18 (2–50.4)
**Landstrom, 2010**^19^	6/6	6/6	4	4	0	1	1	1	0	0	bleo, i.t.	needle, Medpulser	BCC and SCC	biopsy	18.5 (3–24)
**Larkin, 2007**^20^	30/148	26/111	82	64	18	8	8	2	6	13	bleo, i.t or i.v.	plate or needle, Cliniporator	melanoma, SCC, AC, chondrosarcoma	WHO	nd (2–12)
**Matthiessen, 2011**^21^	52/196	24/94	72	57	15	10	4	1	3	8	bleo, i.t or i.v.	plate or needle, Cliniporator	melanoma, SCC, AC, BCC, breast cancer	RECIST	nd (2–6)
**Quaglino, 2008**^11^	14/233	14/233	202	133	69	8	14	3	11	9	bleo. i.v.	plate or needle, Cliniporator	melanoma	WHO	21 (5–28)
**Rols, 2000**^22^	5/61	5/61	24	6	18	34	1	0	1	2	bleo, i.v.	plate, PS 15, Jouan	melanoma, SCC	WHO	1.6 (1–2)
**IO data**	52/379	32/187	131	98	33	44	6	3	3	6	bleo, i.t. or i.v., CDDP, i.t.	plate or needle, Cliniporator	melanoma, carcinoma, sarcoma	WHO	3.2 (1–16)
					
**Summary**	**(%) 253/2226**	**197/1466**	**1040 (85.7)**	**722 (59.5)**	**318 (26.2)**	**174 (14.3)**	**172 (68.2)**	**84 (33.3)**	**88 (34.9)**	**80 (31.8)**					
					
**Summary of all tumors (%)**			**1212 (82.7)**	**806 (55.0)**	**406 (27.7)**	**254 (17.3)**									

OR = objective response (including CR and PR); CR = complete response; PR = partial response; NR = no response (including tumors with no change and progressive disease status); bleo = bleomycin; CDDP = cisplatin; i.t. = intratumoral route of administration; i.v. = intravenous route of administration; BCC = basal cell carcinoma; SCC = squamous cell carcinoma; AC = adenocarcinoma; mo. = month. nd = no data
